# Regional-Scale Drivers of Forest Structure and Function in Northwestern Amazonia

**DOI:** 10.1371/journal.pone.0119887

**Published:** 2015-03-20

**Authors:** Mark A. Higgins, Gregory P. Asner, Christopher B. Anderson, Roberta E. Martin, David E. Knapp, Raul Tupayachi, Eneas Perez, Nydia Elespuru, Alfonso Alonso

**Affiliations:** 1 Department of Global Ecology, Carnegie Institution for Science, Stanford, California, United States of America; 2 Facultad de Ciencias Biológicas, Universidad Nacional de la Amazonía Peruana, Iquitos, Peru; 3 Center for Conservation Education and Sustainability, Smithsonian Conservation Biology Institute, National Zoological Park, Washington, DC, United States of America; Lakehead University, CANADA

## Abstract

Field studies in Amazonia have found a relationship at continental scales between soil fertility and broad trends in forest structure and function. Little is known at regional scales, however, about how discrete patterns in forest structure or functional attributes map onto underlying edaphic or geological patterns. We collected airborne LiDAR (Light Detection and Ranging) data and VSWIR (Visible to Shortwave Infrared) imaging spectroscopy measurements over 600 km^2^ of northwestern Amazonian lowland forests. We also established 83 inventories of plant species composition and soil properties, distributed between two widespread geological formations. Using these data, we mapped forest structure and canopy reflectance, and compared them to patterns in plant species composition, soils, and underlying geology. We found that variations in soils and species composition explained up to 70% of variation in canopy height, and corresponded to profound changes in forest vertical profiles. We further found that soils and plant species composition explained more than 90% of the variation in canopy reflectance as measured by imaging spectroscopy, indicating edaphic and compositional control of canopy chemical properties. We last found that soils explained between 30% and 70% of the variation in gap frequency in these forests, depending on the height threshold used to define gaps. Our findings indicate that a relatively small number of edaphic and compositional variables, corresponding to underlying geology, may be responsible for variations in canopy structure and chemistry over large expanses of Amazonian forest.

## Introduction

Field studies in lowland Amazonia have consistently found relationships at continental scales (100s to 1000s of kilometers) between soil properties, and forest function and structure. Widely-spaced tree plot networks have revealed associations between broad soil fertility classes and trends in tree species functional attributes such as seed mass, wood density, and root nodulation [1]. Plot-based studies have also tied soil drainage and fertility classes to gradients in productivity and aboveground biomass [2–4]. These patterns in forest structure and function have been attributed to a continental-scale gradient from poorer soils on the Brazilian and Guiana shields, supporting slow-dynamic and high-biomass forests, to richer soils in western Amazonia, supporting fast-dynamic and low-biomass forests [1].

At regional scales (10s to 100s of kilometers), however, little is known about the manner in which forest structure and function map onto specific edaphic or geological features. Analyses of Landsat and field data in northwestern Amazonia have revealed discontinuities in plant species composition of hundreds to thousands of kilometers in length, corresponding to geological formations and their edaphic properties [5,6]. These findings have been supported by plot-based research in western Amazonia, which have found relationships between plant composition, and soils or geological properties [5–8]. Most recently, analyses of airborne LiDAR (Light Detection and Ranging) data in southwestern Amazonia have identified patterns in forest biomass and canopy gap distributions, linked to geologic and hydrologic conditions [9,10].

In addition, studies outside Amazonia have identified relationships in tropical forests between geological substrate and soil properties, and forest structure, dynamics, and chemistry. Studies using LiDAR data for the island of Hawai’i have revealed clear differences between basaltic substrates of varying age and nutrient availability in canopy vertical structure, gap frequency, and canopy chemistry [11,12]. Work in Costa Rica and Panama has also uncovered relationships between soil or geological substrate and forest structural parameters [13,14]. Despite these advances, we need more information about regional-scale (10s-100s km) abiotic controls on spatially discrete patterns in forest structure, dynamics, and chemical properties in Amazonian forests. Understanding the relationship between underlying substrate and aboveground forest properties will allow both a better understanding of patterns in these forest properties, and more accurate methods of mapping them.

We used the Carnegie Airborne Observatory (CAO) [15] to generate spatially-explicit maps of forest structure and canopy reflectance for more than 600 km^2^ of forest in northwestern Amazonia. The CAO combines a visible to shortwave infrared (VSWIR) imaging spectrometer with LiDAR to simultaneously measure forest structural and canopy reflectance properties at high spatial resolution (< 2 m). The VSWIR spectrometer collects spectral data in 481 channels of 5 nm bandwidth from the 380 to 2510 nm, allowing measurements of spectral reflectance that are known to be closely tied to canopy chemistry [16–18] and species composition [14,19–21]. The LiDAR provides information on ground elevation and canopy height, as well as the vertical distribution of vegetation at 5 m horizontal resolution, and LiDAR data have been used to study variations in forest structure in a variety of temperate and tropical ecosystems [22–24].

We paired these airborne data with 83 field plots of soils and plant species composition, distributed between two widespread geological formations to study the relationship between, as dependent variables, canopy height, canopy structure, and canopy spectral reflectance; and as independent variables, species composition, soils, and underlying geological formations. We specifically asked: (i) do changes in plant species composition and soil properties translate into variations in forest vertical structure, and what is the strength of the relationship between forest canopy height and soil and compositional variables; (ii) do changes in species composition and soil properties translate into variations in canopy spectra, and what is the strength of the relationship between canopy spectral properties and individual soil and compositional variables; and (iii) how does variability in canopy structure and reflectance correspond to soil properties? We additionally used LiDAR data to calculate the percentage of area occupied by canopy gaps across our study region, and ask (iv) whether gap frequency is related to soil properties. With these data we documented how geological, edaphic, and compositional patterns are translated into forest structure, canopy properties, and dynamics in Amazonian forests at scales of 10s to 100s of kilometers.

## Materials and Methods

### Study area

**Fig 1:**
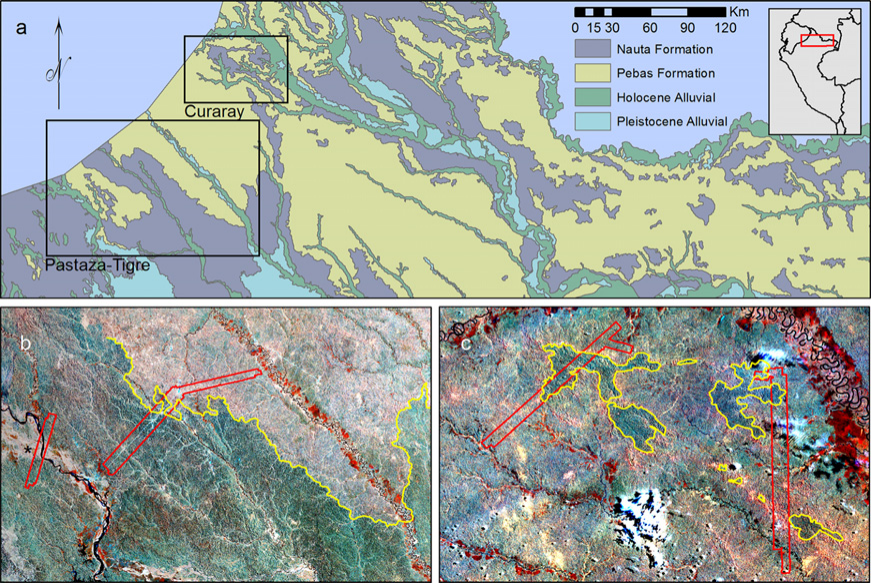
Location of study areas and CAO data in Northern Peru. (a) Location of Pastaza-Tigre and Curaray study areas relative to geological map of Peru [25]. Grey inset shows location of panel relative to outline of Peru. (b) CAO data for Pastaza-Tigre study area, indicated by red lines, superimposed upon Landsat data for study area. Yellow line indicates the boundary between the Pebas Formation (to the west) and the Nauta Formation (to the east). Asterix indicates extension of Pastaza Fan Formation. (c) CAO data for Curaray study area, indicated by red lines, superimposed upon Landsat data for study area. Yellow polygons indicate islands of Nauta Formation surrounded by the Pebas Formation. White patches in the south and northeast of image are clouds. For panels (b) and (c), geological boundaries and Landsat imagery are as described in Higgins [6,26].

We focused our study on two areas in northern Peru containing boundaries between the widespread Pebas and Nauta geological formations (Fig. 1). The Pastaza-Tigre study area consisted of approximately 70 km of road between the Pastaza and Tigre rivers, 30 km northwards along the Pastaza River, and 50 km southwards along the Tigre River. The Curaray study area consisted of an 1800 km^2^ area immediately to the west and south of the Curaray River and was accessed by helicopter during seismic oil exploration. We collected field data for the Pastaza-Tigre study area between 2005 and 2006, and the Curaray study area in 2008. All airborne LiDAR and VSWIR data were collected in 2012. At all sites, we sampled only undisturbed and closed-canopy broadleaf evergreen rainforest. All transects were located in either the Pebas or Nauta Formations, with the exception of three transects in the west of the Pastaza-Fan study area which were located in the volcaniclastic Pastaza Fan Formation (indicated by asterix in Fig. 1b; for more information see [6]).

### Field data collection and processing

We used a combination of Landsat imagery and SRTM (Shuttle Radar Topography Mission) data to identify floristic discontinuities of geological origin, and then sampled these in the field for plant species composition and soils [6]. Tree species inventory in Amazonian forests is notoriously difficult due to large numbers of species, tall and difficult-to-identify individuals, and poorly-known taxonomy [27], and this prohibited the broad-scale but intensive field sampling needed to identify discontinuities in our study area. We thus focused our plant inventories on a single plant group, the pteridophytes (ferns and lycophytes). This group has been used extensively to relate remotely-sensed imagery to patterns in plant composition in Amazonian forests, and is a common tool in the study of biodiversity patterns in western Amazonia [5,8,6,26]. Most importantly, pteridophytes are known to capture a majority of the patterns observed in tree inventories at sites across the Neotropics [5,28–30].

We collected presence-absence data for pteridophytes along 83 linear transects of 5 x 500m (0.25 Ha), consisting of 55 sites at Pastaza-Tigre and 28 sites at Curaray [6]. Inventory sites were located so as to sample the full range of variability observed in Landsat imagery, and such that each transect fell within an area of uniform tone in Landsat imagery based on Landsat bands 4, 5, and 7 [6]. In addition, all transects were restricted to primary forest (i.e. absent anthropogenic disturbance), and were sited so as not to initiate in large treefall gaps. Only individuals with at least one leaf (for ferns) or stem (for lycophytes) longer than 10cm were recorded, and epiphytic and climbing individuals were recorded only if they had green leaves ≤ 2m above ground. We obtained permits for collection and export of plant specimens from the Peruvian National Institute of Natural Resources (INRENA), and deposited vouchers for all species at herbaria in Peru (AMAZ and USM) and Finland (TUR) [31].

We additionally collected soil samples at 50m, 250m, and 450m along each transect. Each of these three soil samples consisted of five subsamples of the top 10cm of mineral soil, collected in an area of 4m x 4m. These five subsamples were combined in the field into a single sample, and equal dry weights of the three samples were combined into one sample per transect for analysis. Soil samples were analyzed at MMT Agrifood (Jokioinen, Finland) for pH; loss on ignition (LOI, a measure of organic matter content); P concentration (Bray method); and extractable Al, Ca, K, Mg and Na (in 1M ammonium acetate). In addition, percentages of sand, silt and clay were determined at MTT Agrifood (Curaray samples; sieving and pipette methods) and the University of Turku Department of Geology (Turku, Finland; laser diffraction). All cation concentrations were log-transformed prior to analysis [6,8]. In addition, for ease of comparison of our soils data to our remotely-sensed data, we used the log-transformed sum of four cations (Mg, Ca, Na, and K) to represent total cation concentrations [5].

We used clustering analysis to visualize compositional patterns in our plant data sets. The Jaccard index was used to calculate distance matrices for both study areas, and unweighted group-mean averaging (UPGMA) to divide the plant inventories into two groups. We additionally used non-metric multidimensional scaling (NMDS) to reduce our floristic data to a single variable for comparison to soils and remotely sensed data. Our species and morphospecies identifications were not standardized between the Curaray and Pastaza-Tigre study areas, and we thus produced separate clustering results and non-metric multidimensional scaling (NMDS) scores for our two study areas.

We calculated one-dimensional NMDS solutions for our plant inventories using the one-complement of the Jaccard index as a distance measure. For this analysis we ran a maximum of 400 iterations from 40 random starting configurations, and applied an instability criterion of 10^5^. All clustering and NMDS calculations were performed with PC-ORD v. 4.41. In addition, we separately calculated NMDS scores for both the full set of 83 transects with LiDAR data and the subset of 74 transects with VSWIR data (see below, “Comparison of CAO and field data”), and used these scores for comparison to the LiDAR and VSWIR data, respectively.

### CAO data collection and processing

We used the CAO Airborne Taxonomic Mapping System (AToMS) [15] to collect co-aligned LiDAR data and VSWIR imaging spectroscopy data for the study areas containing our 83 study sites, yielding a total imaged area of area of approximately 600 km^2^. These data were collected in four blocks of imagery (two per study area) ranging in size from approximately 70 to 340 km^2^. Each block consisted of parallel and overlapping flight lines approximately 1km wide and 30–40 km long. Due to differences between the LiDAR and VSWIR instruments in terms of field-of-view and susceptibility to clouds, the VSWIR footprint was slightly smaller than the LiDAR footprint for all four blocks.

The VSWIR spectrometer produced canopy reflectance data at a spatial resolution of 2 m and a spectral resolution of 5 nm, for a total of 428 channels between 380 and 2512 nm. To reduce data volume and facilitate analysis, these data were resampled to 214 bands of 10 nm width before analysis. Of these we removed 52 bands lying in areas of high atmospheric absorbance, resulting in a total of 162 bands used for analysis. We additionally corrected these data for atmospheric distortions and bidirectional reflectance distribution function (BRDF) effects prior to analysis [32], and omitted bands coinciding to regions of high absorption by water vapor (1320–1500 nm, and 1770–2010 nm). We also removed pixels corresponding to clouds and shaded canopy. Shaded pixels were identified by an algorithm that considered both sun location and height of neighboring pixels derived from the LiDAR data [33]. All VSWIR data processing was performed in ENVI (Version 4.8, Excelis Visual Information Solutions, Virginia, USA).

We used discrete-return LiDAR data to calculate ground elevation at 1 m spatial resolution, top-of-canopy height at 5 m resolution, and canopy vertical profile data at 30 m resolution. From our ground elevation data we additionally calculated degrees slope at 1 m resolution. To calculate ground elevation, we partitioned the LiDAR data into square 100m cells and classified the single lowest-elevation LiDAR return within each cell as ground. We then classified the next nearest returns sequentially, such that if an unclassified return was separated by less than both 1.5 m in elevation and 5.5° from the nearest classified ground return, it was also classified as ground. After all returns were classified, ground returns were used to generate a triangulated irregular network (TIN) which was then converted to a raster surface at 1 m resolution. LiDAR data processing was performed with the LAStools (RapidLasso GmbH, Gilching, Germany) and GDAL (Geospatial Data Abstraction Library, Open Source Geospatial Foundation, http://gdal.osgeo.org) software packages.

To calculate top-of-canopy height, we used the first returns from our LiDAR data, excluding points identified as cloud or birds, to construct a TIN, and converted this to a raster at 1 m resolution. We then subtracted the ground elevation raster from this canopy raster to calculate top-of-canopy height at 1 m resolution. To calculate canopy vertical profile data, we divided the LiDAR data into 30 m cells and each cell into 1m height intervals, with the exception of the 0 to 1 m interval, which was divided into two intervals of which only 0.5 to 1 m was used. We then used all LiDAR returns within each 30 x 30 m area to calculate the percentage of all returns within that interval.

We also used the LiDAR data to calculate the percent area in each transect that was comprised of gaps in the forest canopy. To do this, we used canopy height data to calculate the percentage of pixels in each transect buffer area that contained vegetation less than a specified height, based on nine gap height definitions ranging from < 10 m to <2 m at 1 m increments [34]. Unlike previous studies [10,35] we did not count number of gaps or their sizes, nor did we remove gaps smaller than a certain size. Variations in these percent gap measurements were then compared to variations in both soils and plant species composition (see “Comparison of CAO and field data”, below).

### Comparison of CAO and field data

To compare remotely-sensed and field data, we delineated areas that extended 250m on all sides of each transect (i.e. 250 m buffers with rounded ends; approximately 46 Ha per transect), and used these to calculate mean values for LiDAR and VSWIR measurements, consistent with prior analyses of these transects using Landsat data [26,36]. Prior to these calculations we edited the buffers for each transect to remove clouds and roads. We used these buffers to calculate mean values for the following: ground elevation, terrain slope, top-of-canopy height, reflectance in all 162 VSWIR bands, and per-mil occupancy of vegetation in each height class (i.e. mean vertical profile). Transects with less than half of their area available as LiDAR or VSWIR data were omitted from analysis, resulting in 83 transects available for LiDAR analyses (55 transects at Pastaza-Tigre and 28 at Curaray) and 74 for VSWIR analyses (50 transects at Pastaza-Tigre and 24 at Curaray). These differences in the total number of transects available for analyses with each instrument were caused by differences in the footprints of the two sensors (see above, “CAO data collection and processing”).

We used our vertical canopy profile data to visualize how forest structure varies as a function of both soil cation concentrations and plant species composition. Vertical canopy profiles were plotted as height class versus per-mil number of returns in that class, and compared between either clustering group or cation concentration quartiles (as calculated from the log-transformed sum of Mg, Ca, Na, and K). We also used regression analysis to ask how well canopy height could be explained by soil variables, plant species composition (represented by a single NMDS axis), or elevation and slope. For comparisons of LiDAR data to NMDS scores, we used scores calculated from the full set of 83 transects; and for comparisons of VSWIR data to NMDS scores, we used scores calculated from the subset of 74 transects. Because of nonlinear associations between canopy height, and compositional and environmental variables [37], we used second-order polynomial regressions to model the relationship between canopy height (as the dependent variable) and the remaining variables (as independent variables). We report the goodness of fit of these second order polynomials as R^2^ values. We used our VSWIR, LiDAR, and field data to visualize how canopy reflectance varies in regards to soil properties or plant species composition. Mean spectra for all transects were plotted as wavelength versus percent reflectance, and colored according to clustering group or cation concentration quartiles. We also used regression analysis to measure the relationship between canopy reflectance and soil variables, plant species composition, or elevation and slope. For this we used partial least squares regression (PLSR) to estimate the relationship between all 162 bands of the VSWIR data, and each of the compositional or environmental variables. PLSR is similar to data reduction techniques such as principal components regression or redundancy analysis, in that it allows the reduction of the over 200 bands of the VSWIR data to a linear combination of a smaller number of vectors which can then be compared to environmental and compositional variables. For this reason PLSR is ideally suited to the analysis of imaging spectroscopy data and commonly used for this purpose [38,39]. These analyses were conducted in the JMP statistical package (JMP, Version 10. SAS Institute Inc., Cary, NC, 1989–2007) and the strength of these correlations were reported as R^2^ values.

We also determined how variability in vertical canopy profiles and spectral data changed as a function of plant species composition (NMDS scores) or soil cation concentrations (the log-transformed sum of Mg, Ca, Na, and K concentrations). Variability in canopy height was assessed using the coefficient of variation (CV), which normalizes standard deviation values by the mean value, and variability in spectral reflectance and canopy profiles was assessed using the standard deviation (SD). For canopy height, we calculated the coefficient of variation for each transect, using all pixels in each transect buffer area, and then used polynomial regressions to measure the relationship between cation concentrations (the log-transformed sum of Mg, Ca, Na, and K) and CV (dependent variable) for all transects at each study area. For spectral and profile data, we divided the data into two clustering groups or four soil cation concentrations quartiles, and then calculated the SD for each band or height class based on the mean values for all transects in the clustering or cation group.

Last, we calculated the relationship between soil cation concentrations and the percentage of area at each transect occupied by gap pixels. For this purpose we used polynomial regressions to model the relationship between percent gap as the dependent variable; and cation concentrations (the log-transformed sum of Mg, Ca, Na, and K) or plant species composition (measured by a single NMDS axis), as the independent variables. For this analysis we defined gaps using nine height thresholds, ranging from 2m to 10 m. For each threshold we identified all pixels that fell at or below these thresholds and labelled them as gap. These data were then used to calculate the percentage of each transect occupied by gap.

## Results

### Field data analyses

Our inventories included a total of 149 and 112 species at the Pastaza-Tigre and Curaray study areas, respectively, with an average of 34 and 29 species per transect. Cation concentrations ranged from 0.17 to 24.12 cmol(+)∙kg^−1^ (sum of Ca, Mg, Na, and K) at Pastaza-Tigre and 0.48 to 21.26 at Curaray, comparable to the range observed by previous studies in the region [5,8,36]. These ranges are also within the same order of magnitude as observed at sites across Amazonia [6,8,40–42], indicating substantial variation in soil properties within the two study areas.

**Fig 2:**
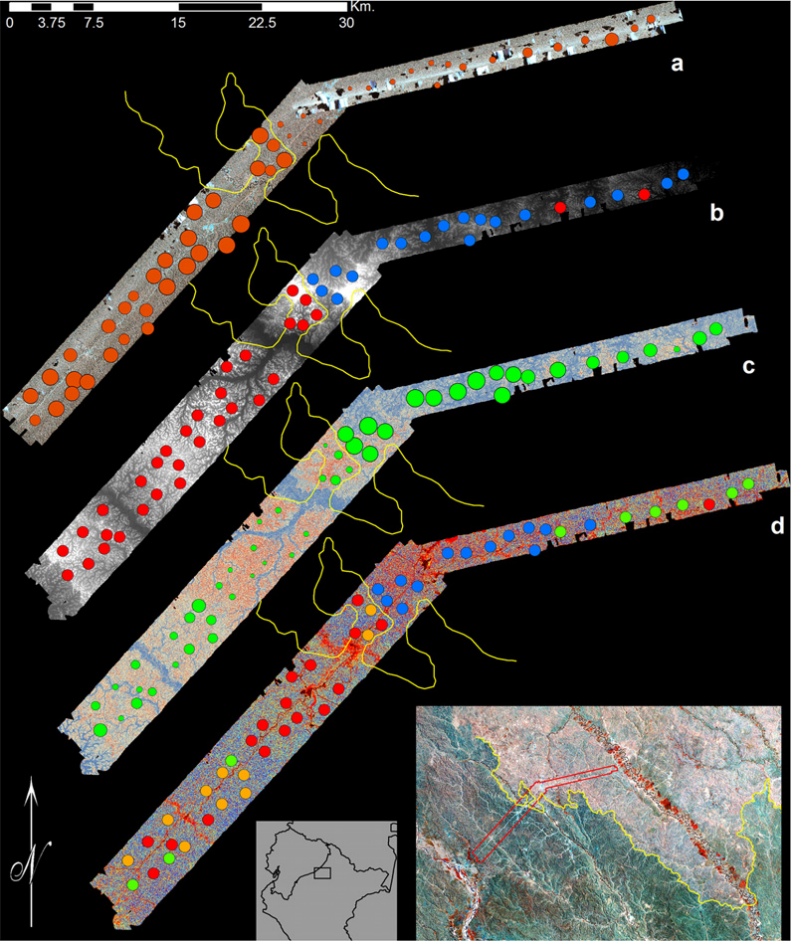
CAO and field data for the eastern half of the Pastaza-Tigre study area. (a) VSWIR data for the eastern half of the Pastaza-Tigre study area, overlaid with NMDS scores for the plant inventories. Imagery represents channels for 830, 1650, and 2220 nm set to red, green, and blue, respectively. Points represent plant inventories, and are sized according to NMDS score. (b) LiDAR ground elevation data overlaid with clustering analysis results. Lighter tones in LiDAR data indicate higher elevations, and darker tones indicate lower elevations. Transect color indicates clustering group (red or blue). (c) Slope data overlaid with cation concentrations. Red tones in the slope data indicate higher slopes, and blue tones lower slopes. Points represent soil samples and are sized by the log-transformed sum of Mg, Ca, Na, and K concentrations. (d) Canopy height data overlaid with quartiles for cation concentrations. Blue tones in the canopy height data indicate higher height, and red tones indicate lower height. Transect color represents its quartile for cation concentrations: Red, first (bottom) quartile; orange, second quartile; green, third quartile; and blue, fourth (top) quartile. In all panels, the yellow line indicates the geological boundary between the Nauta Formation (to the west) and Pebas Formation (to the east). Red outline in large inset indicates the extent of panels (a)-(d), and is overlaid on Landsat data for the study area (as per Fig. 1). Box in small inset indicates the position of the large inset relative to the outline of northern Peru.

Clustering analysis based on our plant data revealed two distinct groups at each study area, divided clearly between the two geological formations (Fig. 2a). This grouping was identical when calculated from both the full set of 83 transects, or the subset of 74 transects for which LiDAR data were available. Based on the full set of 83 transects, 34 transects were associated with the Pebas Formation at the Pastaza-Tigre study area and 21 transects were associated with the Nauta Formation; and 17 transects were associated with the Pebas Formation at the Curaray study area, and 11 transects were associated with the Nauta Formation. The three transects located in the Pastaza Fan Formation at the west of the Pastaza-Tigre study area were classified with the Pebas Formation. The average turnover in species composition between transects from different clustering groups was 88% at Pastaza-Tigre and 80% at Curaray (Jaccard index), corresponding to an average 11-fold and 9-fold change in soil cation concentration (log-transformed sum of Mg, Ca, Na, and K) between the two groups, respectively (Fig. 2c,d). We also observed variation in species composition within the clustering groups but this was substantially less: the average turnover in species composition between transects from the same clustering groups was 57% at Pastaza-Tigre and 58% at Curaray. As previously observed, floristic composition and soil properties are strongly correlated at these sites, making it difficult to tease apart the relative importance of these variables in explaining forest properties [6].

NMDS ordinations of our plant inventory data yielded two independent NMDS axes for each study area: one based on transects with LiDAR data (i.e. the full set of 55 sites at Pastaza-Tigre and 28 sites at Curaray); and one based on transects with VSWIR data (i.e. the subset of 50 sites at Pastaza-Tigre and 24 sites at Curaray; see “Comparison of CAO and field data”, above). These NMDS axes explained 89 and 86% of the variation in the original plant species datasets (i.e. distances between sites as calculated by the Jaccard index) for Pastaza-Tigre and Curaray, respectively (Fig. 2b), regardless of whether the full set of transects with LiDAR data, or the subset of transects with VSWIR data were used for the NMDS calculation.

### Canopy structure

**Fig 3:**
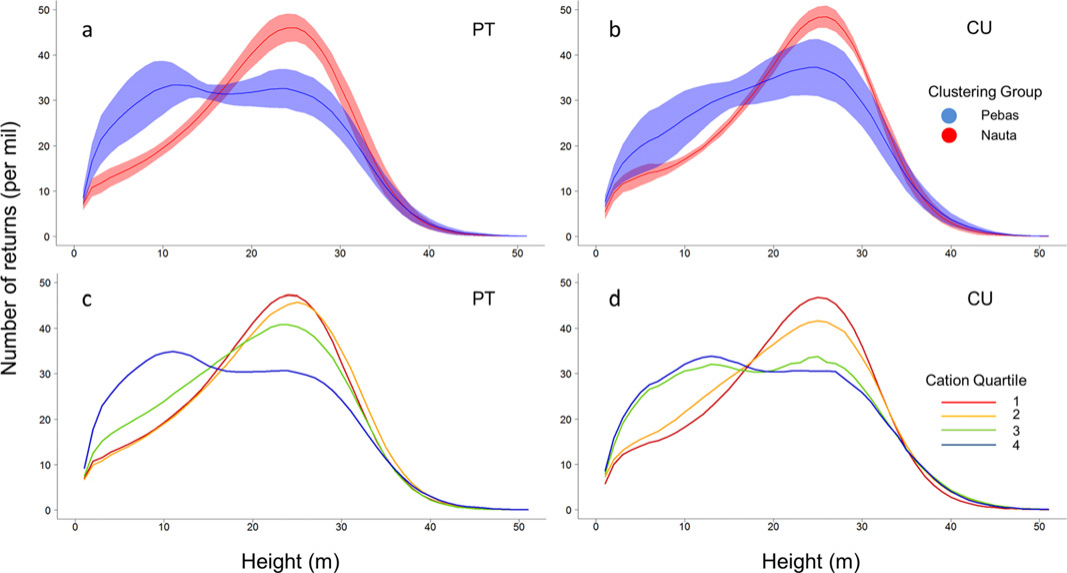
Relationship between canopy vertical structure and floristic composition or soil cation concentrations. Panels display the number of returns (normalized to 1000) for one-meter height classes for transects from the Pastaza-Tigre (“PT”; a,c) or Curaray (“CU”; b, d) study areas. (a, b) Canopy height profile for the Pebas Formation (blue) and Nauta Formation (red) clustering groups, such that color-coding matches Fig. 2b. Blue and red lines indicate the mean profile and semi-transparent areas indicate ±1 standard deviation intervals. (c,d) Mean canopy height profiles for each of the four cation concentration quartiles. Color-coding matches that in Fig. 2d, such that red indicates the first (bottom) quartile, orange the second, green the third, and blue the fourth (top).

The two clustering groups identified from our plant inventories were characterized by substantially different vertical canopy profiles (Fig. 3a, b). Forests growing on the poor soils of the Nauta Formation exhibited a distinct peak in canopy height at approximately 25m, indicating an open understory and a more densely-populated canopy. Forests growing on the rich soils of the Pebas Formation, however, showed a substantial thinning of the 25 m canopy class, and the emergence of a new and less-distinct peak in canopy height at 10 m, indicating a more densely-populated understory and open canopy.

Comparison of these structural differences to soil cation concentrations revealed a gradient in forest structures between these two extremes (Fig. 3c,d). At the lowest quartile of cation concentrations, vegetation was concentrated in the 25 m height class with substantially less vegetation in larger and smaller height classes. Increased soil cation concentrations resulted in a progressive shift of vegetation from the 25 m height class to the 10 m height class, with little apparent increase in intermediate height classes (e.g. 15m). These findings indicate a proportional shift of vegetation structure from the canopy to understory as cation concentrations increase.

These relationships between species composition or soils, and forest structure, were confirmed by regression analysis. NMDS scores, Mg concentrations, and Ca concentrations were the three variables most strongly correlated with canopy height at Pastaza-Tigre, where they explained approximately 70% of the variation in canopy height (Table 1). NMDS, Mg, and Ca were also three of the four most strongly correlated variables at Curaray (in addition to pH), where they explained approximately 40% of the variation in canopy height. After pooling our soils data from both study areas, Mg and Ca concentrations were the two most strongly correlated variables overall, and were able to explain approximately 60% of the variation in vegetation height. Overall, average canopy height was 14% and 9% greater on the poor soils of the Nauta Formation, at Pastaza-Tigre and Curaray respectively (24.1 m and 21.1 m on the Nauta and Pebas Formations, respectively, at Pastaza-Tigre; and 25.2 and 23.2 at Curaray). In general, the correlations for the pooled data were intermediate for the correlations for the individual sites, with the notable exception of slope, which was substantially more poorly correlated with canopy height for the pooled data. This is because slope exerts an opposite effect at the two study areas, and is positively correlated with height at Pastaza-Tigre and negatively correlated at Curaray.

**Table 1:** Relationship (R^2^) between vegetation height and compositional or environmental variables as calculated by second-order polynomial regressions, for Pastaza-Tigre (PT), Curaray (CU), or both areas combined (All).

Variable	PT	CU	All
NMDS*	0.74	0.42	-
Log Mg (cmol^+^ kg^-1^)	0.75	0.40	0.60
Log Ca (cmol^+^ kg^-1^)	0.69	0.41	0.60
pH	0.46	0.48	0.46
Log K (cmol^+^ kg^-1^)	0.40	0.34	0.36
Log P (cmol^+^ kg^-1^)	0.35	(0.19)	0.32
Log Al (cmol^+^ kg^-1^)	0.28	0.37	0.29
Elevation (m)	0.22	(0.23)	0.20
Slope (°)	0.60	(0.24)	0.19
Clay (%)	0.23	(0.20)	0.16
Silt (%)	0.23	(0.21)	0.16
Sand (%)	0.25	(0.03)	(0.06)
Log Na (cmol^+^ kg^-1^)	0.17	(0.27)	(0.04)
LOI (%)	0.18	(0.06)	(0.03)

* Comparison of NMDS scores to vegetation height for both study areas combined (“All”) was not possible as taxonomy was not standardized between sites

All values significant at P < 0.01 unless indicated by parentheses, and values sorted by results for both study areas.

### Canopy reflectance

**Fig 4:**
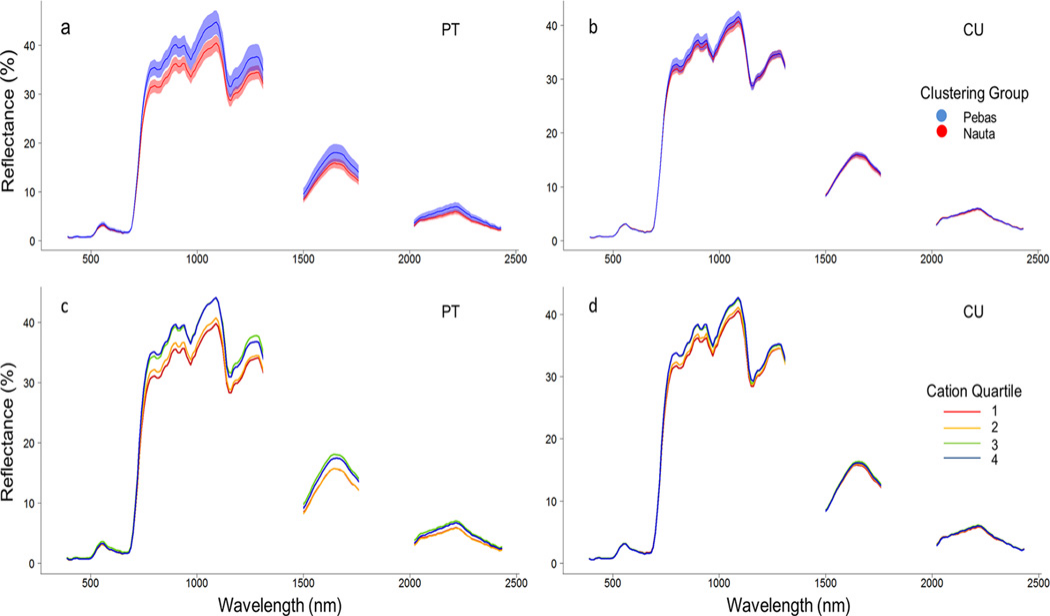
Relationship between canopy reflectance and floristic composition or soil cation concentrations. Panels display reflectance spectra for transects from the Pastaza-Tigre (“PT”; a,c) or Curaray (“CU”; b,d) study areas. (a, b) Mean spectra for transects from the Pebas Formation clustering group (blue) or Nauta Formation clustering group (red), such that color-coding matches Fig. 2b. Blue and red lines indicate mean spectra for all transects in the clustering group, and semi-transparent areas indicate ±1 standard deviation intervals. (c,d) Mean spectra for each of the four cation concentration quartiles. Color-coding matches that in Fig. 2d, such that red indicates the first (bottom) quartile, orange the second, green the third, and blue the fourth (top).

The clustering groups identified from our plant inventories were also characterized by distinct reflectance spectra (Fig. 4a,b). In both study areas, forests growing on the Pebas Formation exhibited higher reflectance relative to the Nauta Formation, and this was most pronounced in the near infrared (NIR). Classification of these spectra by soil cation concentrations (the log-transformed sum of Mg, Ca, Na, and K) revealed an increase in reflectance from the poorest quartile to richest quartile, indicating that canopy reflectance is correlated with edaphic properties (Fig. 4c,d). The separation in spectra between the clustering groups and cation quartiles was most pronounced in the near infrared (i.e. 750 to 1150 nm), consistent with recent findings from Landsat data [26].

These relationships between plant species composition and soil properties, and canopy reflectance, were confirmed by PLSR analyses (Table 2). NMDS scores explained 94 and 95% of the variation in canopy reflectance at Pastaza-Tigre and Curaray, respectively, and was the strongest predictor of reflectance at Pastaza-Tigre. Most soil variables explained 85% or more of the variation in reflectance at both study areas, and due probably to this high percent of explained variation, the rankings of soil variables at the two study areas was not consistent. After pooling data from both areas, however, log-transformed Mg and Ca concentrations emerged as two of the three most important variables following elevation, consistent with our findings for canopy height.

**Table 2:** Relationship (R^2^) between spectral data and compositional or environmental variables, as calculated by PLS regression, for Pastaza-Tigre (PT), Curaray (CU), or both areas combined (All).

Variable	PT	CU	All
NMDS *	0.94	0.95	-
Elevation (m)	0.85	0.96	0.93
Log Mg (cmol+ kg-1)	0.90	0.93	0.89
Log Ca (cmol+ kg-1)	0.88	0.93	0.88
Log K (cmol+ kg-1)	0.88	0.94	0.83
Veg. height (m)	0.85	0.94	0.83
Slope (°)	0.85	0.89	0.82
pH	0.91	0.93	0.79
LOI (%)	0.85	0.98	0.79
Log Al (cmol+ kg-1)	0.88	0.95	0.78
Clay (%)	0.68	0.94	0.77
Sand (%)	0.65	0.96	0.65
Log Na (cmol+ kg-1)	0.52	0.97	0.63
Silt (%)	0.65	0.95	0.58
Log P (cmol+ kg-1)	0.75	0.93	0.57

* Comparison of NMDS scores to vegetation height for both study areas combined (“All”) was not possible as taxonomy was not standardized between sites

All values significant at P < 0.01, and values sorted by results for both areas.

### Variability in canopy structure and reflectance

**Fig 5:**
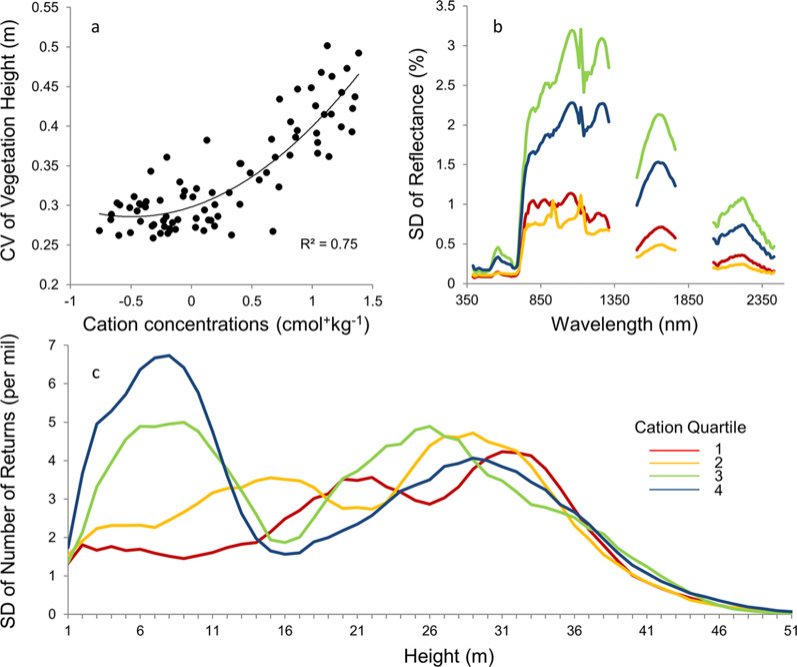
Relationship between cation concentrations and variability in canopy structure and reflectance. (a) Relationship between soil cation concentrations (the log-transformed sum of Mg, Ca, Na, and K) and coefficient of variation for canopy height. Trend line represents second-order polynomial. (b,c) Average standard deviation for canopy spectra and vertical height profiles, respectively, after grouping by cation concentration quartiles. In (b) and (c), color-coding matches that in Fig. 2d, such that red indicates the first (bottom) quartile, orange the second, green the third, and blue the fourth (top).

In addition to strong relationships between soil cation concentrations, and forest structural and reflectance variables, we also found relationships between soils and the degree of variation in these variables (Fig. 5). Increased cation concentrations resulted in a doubling of the coefficient of variation for vegetation height, such that transects on poorer soils were more uniform in height and transects on richer soils were more variable in height. Pooling data for both study areas, cation concentrations explained 75% of the variability in canopy height observed within individual transects (Fig. 5a).

This variability in forest structure was also expressed as variability in individual height classes. The greatest variability in vegetation density was observed in smaller height classes (1 to 15 m) on the richest quartile of soils (Fig. 5c; blue line). As cation concentrations decreased (Fig. 5c; green to yellow to red lines), however, variability in vegetation density in small height classes decreased and variability increased in taller height classes (e.g. from peaks in variability at approximately 26, 29, and 33 m, moving from the second-highest to lowest quartile). Variability in the mid-canopy (i.e. 15m) was least affected by cation concentrations, consistent with the absolute values reported for canopy height. In all these cases, variability on poorer soils at any height class did not exceed that observed in the smallest size classes on rich soils.

This increase in structural variability on richer soils was paralleled by increased variability in canopy reflectance (Fig. 5b). On average, variability in reflectance was highest for spectra in the two richest quartiles of cation concentrations, and lowest for spectra in the two poorest quartiles, and these differences were observed across all wavelengths. The relative position of the spectra within the two richest and poorest quartile, however, varied by wavelength.

### Gap frequency

**Fig 6:**
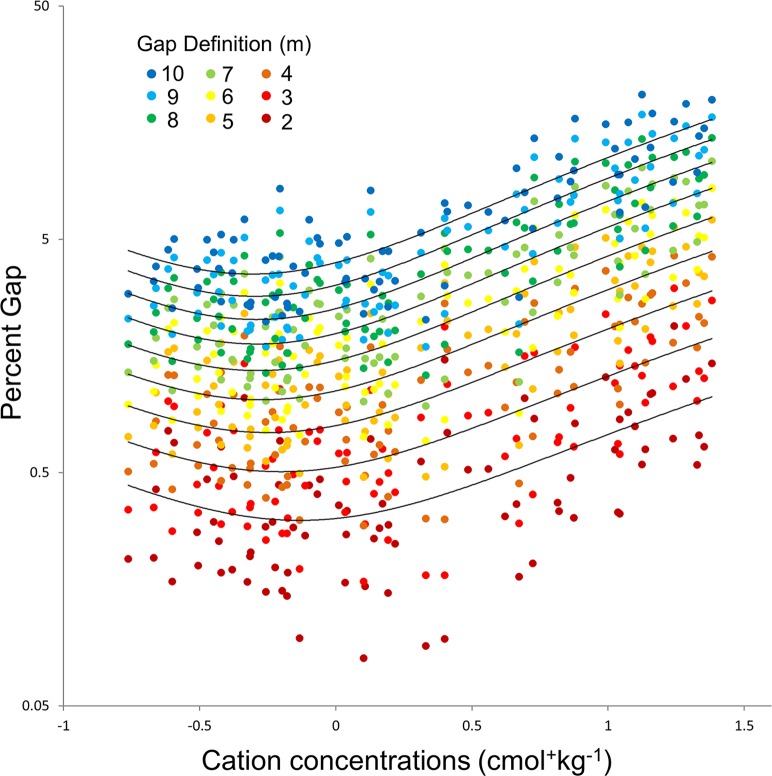
Relationship between gap frequency and cation concentrations for nine gap definitions. Points indicate the percentage of transect area occupied by gap pixels for nine gap definitions ranging from 10 m (blue tones) to 2 m (red tones), at 1m intervals. Lines represent second-order polynomial models for percent gap area based on cation concentrations (the log-transformed sum of Mg, Ca, Na, and K) in descending order from top to bottom from 10 to 2 m, and have an R^2^ value of 0.71, 0.70, 0.69, 0.68, 0.65, 0.62, 0.56, 0.47, 0.35, respectively. Y axis is log10 transformed.

We found significant relationships between percentage of area in gaps and soil cation concentrations for all gap height definitions (Fig. 6), such that increases in cation concentrations between our poorest and richest sites corresponded to an order of magnitude increase in gap frequency, regardless of gap height definition. The relationship between gap percentage and soils was best modeled as a second-order polynomial, such that gap area was lowest at low cation concentrations and increased rapidly at higher concentrations, but also exhibited a slight increase in the lowest cation concentrations. The model of the relationship between soils and gap frequency was essentially identical for all gap height definitions, but displaced on the Y-axis due to the smaller total area in gaps at more strict (i.e. shorter) gap height definitions. The strength of the relationship between percent gap and soils also declined monotonically with the gap height definition, such that the correlation between percent gap and soils was weakest for the shortest (i.e. 2m) gap height threshold and strongest for the tallest threshold (i.e. 10m). We attribute this to the increasingly small number of gaps under the more restrictive gap definitions, resulting in a lower sample size for smaller gap classes (e.g. <2 m) and a less strong fit to the statistical model.

## Discussion

Using a combination of airborne imaging spectroscopy, LiDAR, and field data, we found discrete patterns in forest vertical structure and canopy reflectance in northwestern Amazonia, mapping closely to underlying patterns in soil fertility, plant species composition, and geology. Soil cation concentrations and plant species composition explained up to 70% of the variation in canopy height, and up to 90% of the variation in canopy reflectance. These patterns were expressed as changes in forest vertical structure, from taller, uniform-height, and closed-canopy forests on poorer soils; to shorter, variable-height, open canopy forests on richer soils. In all cases, these patterns in height and structure corresponded to boundaries between underlying geological formations. These findings fill a hole in our knowledge about the possible regional controls on forest structure and function in Amazonia, and suggest that a relatively small number of edaphic and compositional variables may be responsible for variations in canopy height, structure, and chemistry over tens to hundreds of kilometers in Amazonian forests. At broader scales, we anticipate that soil properties and underlying geology will provide a template for forest structural and functional variation, upon which other variables such as climate and distance may be superimposed.

In addition to demonstrating the link at regional scales between soils and forest structure and function, our data paint a picture of two forest types, separated by as little as half a kilometer yet varying significantly in structure, dynamics, and chemistry. Forests on the poor soils of the Nauta Formation were tall and even in height, while forests on the rich soils of the Pebas Formation were substantially shorter and mixed-height. Moreover, forests on the Nauta Formation were relatively invariable in height and canopy reflectance, suggesting a stable and limited set of structural and chemical traits; while forests on the Pebas Formation were substantially more variable, suggesting a broader range of structural and chemical traits. In addition, gaps were significantly less common on the poorer Nauta Formation, and more abundant on the rich soils of the Pebas Formation.

One possible interpretation of our findings is that low rates of gap formation on the less fertile Nauta Formation have resulted in a relatively simple forest structure and less variable set of chemical traits; and that high rates of gap formation on the more fertile Pebas Formation have resulted in highly variable canopy structure and a broader array of chemical traits. This interpretation is consistent with relationships observed elsewhere in the tropics between geologic substrate age and fertility, and canopy structure, chemistry, and gap dynamics [11–13]. They are also consistent with the slower dynamics, higher wood densities, and lower mortality observed on poorer soils in Brazil versus richer soils in western Amazonia [1–3,43,44]. Given the broad distributions of these two geological formations in northwestern Amazonia we expect these patterns to be widespread (Fig. 1) [25].

Between these extremes, we observed a gradient in soils and plant species composition, corresponding to a gradient in the structural and functional properties described above (Fig. 2; see also [6]). As such, though the boundaries between these geological formations and forest types are distinct, we observed variation in species composition, soils, and canopy structure and chemistry within these formations. This is consistent with recent reports from southern Peru finding a strong relationship between these variables at substantially finer spatial scales [45], indicating that the influence of soils upon forest structure and chemistry may exhibit nested properties: from local topographic variation (10s of meters), to landscape scale variations in soils and drainage (10s of meters to 10s of kilometers), to regional-scale variation between geological formations (10s to 100s of kilometers). We furthermore expect that reductions in cation concentrations beyond those observed here may result in reduced canopy height due to reductions in growth rates, consistent with white sand forests several hundred kilometers distant [46]. This reduction on growth rates may explain the apparent slight increase in gap frequency observed at the lowest cation concentrations, due to increased time required to fill gaps.

Our findings come with two caveats. First, the strength of the relationship between soils or plant species composition, and canopy structure and reflectance, appears to depend on the degree of edaphic variation in the study area, as illustrated by the differences between our two study areas. The range of cation concentrations at the Pastaza-Tigre study area is approximately 15% greater than at the Curaray study area, due primarily to the absence of very poor soils at Curaray. Thus, though the relative differences between forests growing upon the Pebas and Nauta Formation are the same at both study areas, the absolute differences in canopy height and spectra are greater. These smaller differences between sites at Curaray may result in less clear patterns relative to background stochasticity, and thus lower correlation coefficients between soils or composition, and canopy height and reflectance (Tables 1, 2; Fig. 4b,d). Possible explanations for the smaller edaphic range at Curaray may include the smaller size of the Curaray study area, fewer transects, or differences between the Nauta Formation deposits at the two study areas. In any case, these differences do not affect the fundamental contrast between forests growing on these two geological formations.

Second, the relationship between canopy spectroscopic and chemical properties cannot be established for a specific study site without field measurements of canopy chemistry, and we were not able to access the canopies on the ground to make a quantitative spectral-to-chemical connection. This said, the differences we observed in canopy spectral reflectance properties between the Nauta and Pebas Formations are strongly suggestive of chemical differences between the forests on these formations (Fig. 4). Numerous studies have previously linked leaf and canopy spectroscopy to canopy chemistry [47–49], and in light of the pronounced differences between these two geological formations and previous studies in tropical forests, we are confident that these spectral differences reflect changes in canopy chemistry.

Specifically, canopy reflectance on the richer soils of the Pebas Formation was greater at all wavelengths than on the poorer soils of the Nauta Formation (Fig. 4), consistent with observations from Landsat data for northern Peru and spectroscopic data for Panama [14,26,36]. The difference between spectra for Pebas and Nauta Formations forests was most conspicuous in the near infrared (800 to 1100 nm), also consistent with findings from Landsat data for this region, suggesting significant differences in total leaf area volume or LAI [26,50]. These formations also differed, but less clearly, in reflectance in shortwave infrared regions 1 and 2 (1500 to 1800 nm, and 2100 to 2400 nm, respectively), suggesting higher leaf mass per area (LMA), leaf water concentration, and lower defense compound chemical investment on the Pebas Formation than the Nauta Formation [18]. An alternate but interesting explanation for these differences might be a greater epiphyte load on leaves on the Nauta Formation, due possible to lower rates of leaf turnover, resulting in increased NIR absorption and reduced reflectance [51]. Transects from the two formations were generally inseparable in the visible wavelengths, however, indicating similar investments in chlorophyll and nitrogen concentrations in foliage [48,49].

Last, our findings last suggest a role for plant species composition in regulating forest structure and function in lowland Amazonia. Plant species composition, as measured by a single NMDS axis, was as strong a determinant as soil cation concentrations of all variables measured here, including canopy height, forest vertical structure, and canopy reflectance. This suggests that composition may be as important as soils in controlling forest properties, and that the effect of soils on canopy structure, chemistry, and dynamics might be mediated by changes in plant species composition. At both study areas, however, soil properties and plant species composition are highly correlated, making it difficult to determine whether variations in plant species composition are determinants of variations in forest structural and functional properties, or independent and unrelated consequences of changes in soil properties. Furthermore, underlying variables such as local topography, fluvial history, and geology are also known to be correlated with both soils and species composition [5,8], raising the possibility that these may independently be responsible for variations in forest structure and canopy properties, rather than soils or species composition. These uncertainties underline the importance of further study and analysis of these remote and globally-important forests.

## Supporting Information

S1 Dataset Data used for analyses of Pastaza-Tigre sites. Center X and Y coordinates are reported in UTM Zone 18S, WGS1984.
(XLSX)

S2 Dataset Data used for analyses of Curaray sites. Center X and Y coordinates are reported in UTM Zone 18S, WGS1984.
(XLSX)
